# Three-dimensional bioprinted in vitro glioma tumor constructs for synchrotron microbeam radiotherapy dosimetry and biological study using gelatin methacryloyl hydrogel

**DOI:** 10.1038/s41598-025-88793-9

**Published:** 2025-04-22

**Authors:** John Paul O. Bustillo, Elette E.M. Engels, Vincent de Rover, Kiarn Roughley, Julia Rebecca D. Posadas, Elrick T. Inocencio, Danielle Warren, Gordon G. Wallace, Moeava Tehei, Anatoly B. Rosenfeld, Michael L.F. Lerch

**Affiliations:** 1 Centre for Medical Radiation Physics, University of Wollongong Australia, Wollongong, NSW 2522 Australia; 2 Department of Physical Sciences and Mathematics, College of Arts and Sciences, University of the Philippines Manila, Ermita, Manila City, Metro Manila, 1000 Philippines; 3 Imaging and Medical Beamline, Australian Nuclear Science and Technology Organisation- Australian Synchrotron, Kulin Nation, Clayton, VIC 3168 Australia; 4 Department of Radiology, University of the Philippines- Philippine General Hospital, Metro Manila, 1000 Philippines; 5 AIIM Facility, Intelligent Polymer Research Institute, ARC Centre of Excellence for Electromaterials Science, University of Wollongong, Wollongong, NSW 2522 Australia

**Keywords:** Synchrotron Radiation, Microbeam Radiation Therapy, Spatial fractionation, Bioprinting, Glioma, GelMA, 3D Printing, Biofabrication, Brain Cancer, Radiotherapy, CNS cancer, Translational research, Techniques and instrumentation, Biomedical engineering, Biophysics

## Abstract

Synchrotron microbeam radiotherapy (MRT) is an innovative cancer treatment that uses micron-sized of ultra-high dose rate spatially fractionated X-rays to effectively control cancer growth while reducing the damage to surrounding healthy tissue. However, the current pre-clinical experiments are commonly limited with the use of conventional two-dimensional cell cultures which cannot accurately model in vivo tissue environment. This study aims to propose a three-dimensional (3D) bioprinting gelatin methacryloyl (GelMA) hydrogel protocol and to characterize 3D bioprinted glioma relative to cell monolayer and spheroid models for experimental MRT using 9L rat gliosarcoma and U87 human glioma. Synchrotron broad-beam (SBB) and MRT beams were delivered to all cell models using 5, 10, and 20 Gy. 3D bioprinting enables the creation of 3D cell models that mimic in vivo conditions using bioinks, biomaterials, and cells. Synchrotron dosimetry, Monte Carlo simulation, in vitro cell viability, and fluorescence microscopy were performed to understand the relationship of the radiation dosimetry with the radiobiological response of different cancer models. Encapsulated gliomas were placed inside 3D printed human and rat phantoms to mimic scattering conditions. Results showed that MRT kills more gliomas relative to SBB for all cell models. The 3D bioprinted culture detected the spatial clustering of dead cells due to MRT high peak doses as seen in fluorescence imaging. The result of this study progresses MRT research by integrating 3D bioprinting techniques in radiobiological experiments. The study’s bioprinting protocol and results will help in reducing the use of animal experiments and possibly in clinical translation of MRT.

## Introduction

Glioma is the most common variant of central nervous system (brain and spinal cord) neoplasm originating from glial cells and considered as one of the most lethal cancers. The most malignant type of glioma is glioblastoma multiforme (GBM) which is often resistant to conventional cancer treatments^1,2^. Glioma treatment modalities consist of surgery, chemotherapy, immunotherapy, and radiation therapy wherein patients’ survival outcome depends on the glioma variant and prognostic factors. Radiation therapy is an important treatment for GBM post-surgery to destroy residual GBM cells at the resection area to avoid cancer relapse^3,4^.

Radiotherapy has been one of the modalities useful in treating brain tumors that aims to reduce the tumor size and the pressure inside the patient’s head. Whole-brain radiotherapy (WBRT) is a common X-ray cancer treatment technique considering the possible metastasis due to tumor cell migration. However, WBRT is not an ideal option in treating primary tumors as they could affect the brain functionalities such as its cognitive processes^5^. The mechanism and understanding of brain radiation toxicity and cognitive deterioration are still incomplete and an active research field^6,7^. Improvements in radiation therapy such as intensity modulated radiation therapy and proton therapy give a significant reduction in normal tissue dose by limiting the dose to the peritumoral treatment volume. Furthermore, the reduction of cranial radiation exposure will decrease the possibility of cognitive deficits (i.e. difficulty in thinking and concentrating) which is one of the treatment’s negative concerns^8^. Regardless of these developments in the current brain cancer treatment, tumor radioresistance and normal tissue tolerance limit the effectiveness of radiotherapy^9,10^.

Synchrotron radiation is generated by charged particles accelerated at relativistic speed in the presence of a strong magnetic field produced by the synchrotron insertion device such as undulator and wiggler. This type of radiation has a broad spectrum, high intensity, and quasi-parallel beam. Beam filters are used to increase the energy of the radiation spectrum and to reduce the entrance dose rate of the beam^11^. Microbeam radiation therapy (MRT) is an innovative synchrotron treatment beneficial in treating animal brain tumors^10^. MRT uses the properties of synchrotron radiation passing through a multi-slit collimator (MSC) creating spatially fractionated high-dose rate of synchrotron kilovoltage X-ray beams at micrometer range which has a potential in improving the therapeutic ratio. MRT beams are described by narrow high dose rate peaks which are separated by low dose-rate valleys produced due to scattered radiation. It commonly has a width around 25–100 μm and a pitch of 100–400 μm depending on the MSC design. Without the MSC from the synchrotron radiation beam path, the uncollimated radiation field called synchrotron broad-beam (SBB) is produced^12^. This unique treatment modality is related to the established experiences in other modalities of spatial fractionated radiotherapy (SFRT) such as GRID and lattice-based radiotherapy. SFRT involves spatially modulated doses creating high and low dose regions, for example MRT and minibeam radiotherapy.

The mechanisms explaining MRT effectiveness are still under research which mainly involve various biological processes such as bystander effect, vascular, and immune response^13^. The synchrotron radiation has a high brilliance, low divergence that can be delivered in thousands of Gray per second (Gy/s) dose rate. Relative to conventional clinical linear accelerator (Linac) megavoltage (MV) X-ray beam WBRT, MRT has radiobiological advantage called dose-volume effect as the normal tissue dose tolerance is higher for small X-rays fields. Thus, it is possible to achieve tumor control in the brain while not affecting the brain functions^12,14^. Due to the high dose rates available in a synchrotron facility, FLASH effect may be observable for both SBB and MRT modes. FLASH effect is defined as a reduction to normal tissue damage while causing similar anti-tumor effect at isodose as conventional radiotherapy dose rate^15^. The MRT pre-clinical trial can be progressed further by incorporating patient-specific methods in studying the dosimetry and three-dimensional in vitro cell response.

Additive manufacturing (AM) or 3D printing was first introduced in the 1980s and has been used in various patient-specific medical applications. In radiological imaging and radiotherapy, AM has been utilized in fabricating polymer-based phantoms and bolus for clinical Linac^16–20^, pre-clinical research quality assurance^21,22^, and in fabricating breast imaging phantom for synchrotron computed tomography^23,24^. Various AM materials have been comprehensively characterized in previous studies for synchrotron radiation micro-computed tomography (CT) imaging and SBB radiation therapy applications^25,26^. A specific subfield of AM called 3D bioprinting is a multidisciplinary area combining rapid manufacturing techniques with bioengineering in fabricating 3D biological construct through layer-by-layer positioning of a bioink composed of biomaterials and cells. This is advantageous relative to conventional in vitro cell culture due to its more realistic simulation of an actual 3D cell environment and possibly a more patient-specific treatment in tissue engineering. Some of the main technologies utilized in bioprinting are micro extrusion, inkjet, and laser-assisted printing^27^. There are few radiation-related studies that attempted to use bioprinting technologies in radiotherapy research in comparison with the traditional two-dimensional (2D) in vitro experiments^28,29^.

A 2D cell culture is commonly used in most neural cancer tissue research, especially for radiotherapy applications. However, 2D culture is very limited when compared to the three-dimensional (3D) characteristics of in-vivo cells surrounded by extracellular matrix (ECM) with established cell-to-cell communication and adhesion. Results in 2D cell monolayer models may not be clinically relevant. ECM is mainly composed of various macromolecules. It is significant in controlling cell growth and extracellular communication. With the current developments in bioengineering, 3D biomaterials capable of simulating the 3D cell culture condition are now available^30^. A published study shows that 3D bioprinted glioma mimics the tumor microenvironment (TME) as it shows radiation resistance due to an increase in ITGA2 genes^31^. Another study co-cultured a human GBM cell line (U251) with endothelial cells (HUVECs) shows an improvement in DNA repair with lower DNA double-strand breakage relative to GBM monolayer culture^32,33^. A bioprinted GBM on-a-chip has been studied as well for the treatment response of combining temozolomide (TMZ) chemotherapy and radiotherapy^34^. However, the inherent mechanisms in using 3D bioprinted brain cancer constructs under synchrotron MRT research are not yet well understood.

Three-dimensional bioprinting offers a significant advancement producing in vitro 3D cancer models with a more accurate and reproducible biological environment relative to conventional two-dimensional cancer cell culture^35^. This technology has a potential in reducing the use of animals in pre-clinical trials for cancer radiotherapy studies. 3D bioprinted glioblastoma model done by previous research has been shown to be an excellent technique in studying novel cancer treatment modalities considering the accurate cellular crosstalk and TME^36,37^. Although 3D bioprinted constructs are not the same as a real organ or tumor, it can simulate the structure and physiological response due to the 3D spatial interaction of cells with its surrounding cell matrix^38^. 3D bioprinting can reduce animal model experiments with its capability to mimic realistic 3D cell culture environment. This is align with the objectives of the New South Wales, Australia’s Non-Animal Technologies Network (NAT-Net) in replacing and reducing the use of animals in medical research by using 3D bioprinted constructs, organoids and tumoroids.

This study aims to provide new understanding on the in vitro cell response of 3D bioprinted glioma constructs to MRT and its correlation with dosimetry. The fabricated 3D bioprinted constructs were incorporated in both standard phantoms and 3D printed phantoms to simulate an actual radiation treatment scenario. This pilot study demonstrated important radiobiological responses after delivering synchrotron X-ray radiotherapy beams to 3D cell cultures. Results of the study can be a basis for future medical physics bioprinting research in designing a more patient-specific X-ray quality assurance dosimetry considering both the patient’s anatomy (3D printed phantoms) and the radiobiological response (3D bioprinted constructs)^39^. Future studies can use the presented methodology in co-culturing cancer and healthy cells using 3D bioprinting techniques to investigate their response after treatment delivery and in studying radiotherapy FLASH effects.

## Materials and methods

### Bioprinting protocol

#### Bioink preparation and optimization

Hydrogel is a 3D network of hydrophilic polymers useful in tissue-engineering to provide an artificial ECM for cells. It is known for its suitable physical and biological properties in cellular microenvironment. In 3D bioprinting, biocompatibility of hydrogel should be considered important for cell proliferation and in forming tissue construct. Gelatin methacyloyl (GelMA) is a semi-synthetic hydrophilic hydrogel created through a covalent bond between gelatin and methacrylic groups. Important features of this hydrogel are biocompatibility, cell adhesion, and customizable mechanical properties^40,41^. GelMA has been investigated as a possible alternative to protein-based hydrogels such as Matrigel®for biological applications and 3D cell cultures. It can be photo-polymerized using a free radical-based crosslinking through a photoinitiator molecule via excitation using a particular wavelength of light^40,42^. Due to its mechanical stability and superior biocompatibility as reported in literature, this pilot study at the Australian synchrotron used GelMA as the main hydrogel in 3D bioprinting^43,44^.

Sterilized lyophilized GelMA with 82 ± 4% degrees of functionalization (DoF) manufactured using gelatin from porcine skin (Translational Research Initiative for Cellular Engineering and Printing, TRICEP^™^, B/N 051022st4) was used in preparing the bioinks of this study. The storage modulus of GelMA varies depending on the animal source (i.e. porcine skin, bovine skin, and fish skin), and on the crosslinking temperature. The use of high concentrations of GelMA and high DoF is recommended in bioprinting by published studies. Porcine derived GelMA with high degree of substitution showed to have higher mechanical strength in coordinated crosslinking. Detailed comparison of GelMA from different animal sources can be found in published studies^45,46^. Due to GelMA tunable mechanical characteristics and biocompatibility, it has good printability for 3D bioprinting applications. However, this should be balanced with the cell proliferation which can be negatively affected by the polymer network density^47,48^.

**Fig. 1:**
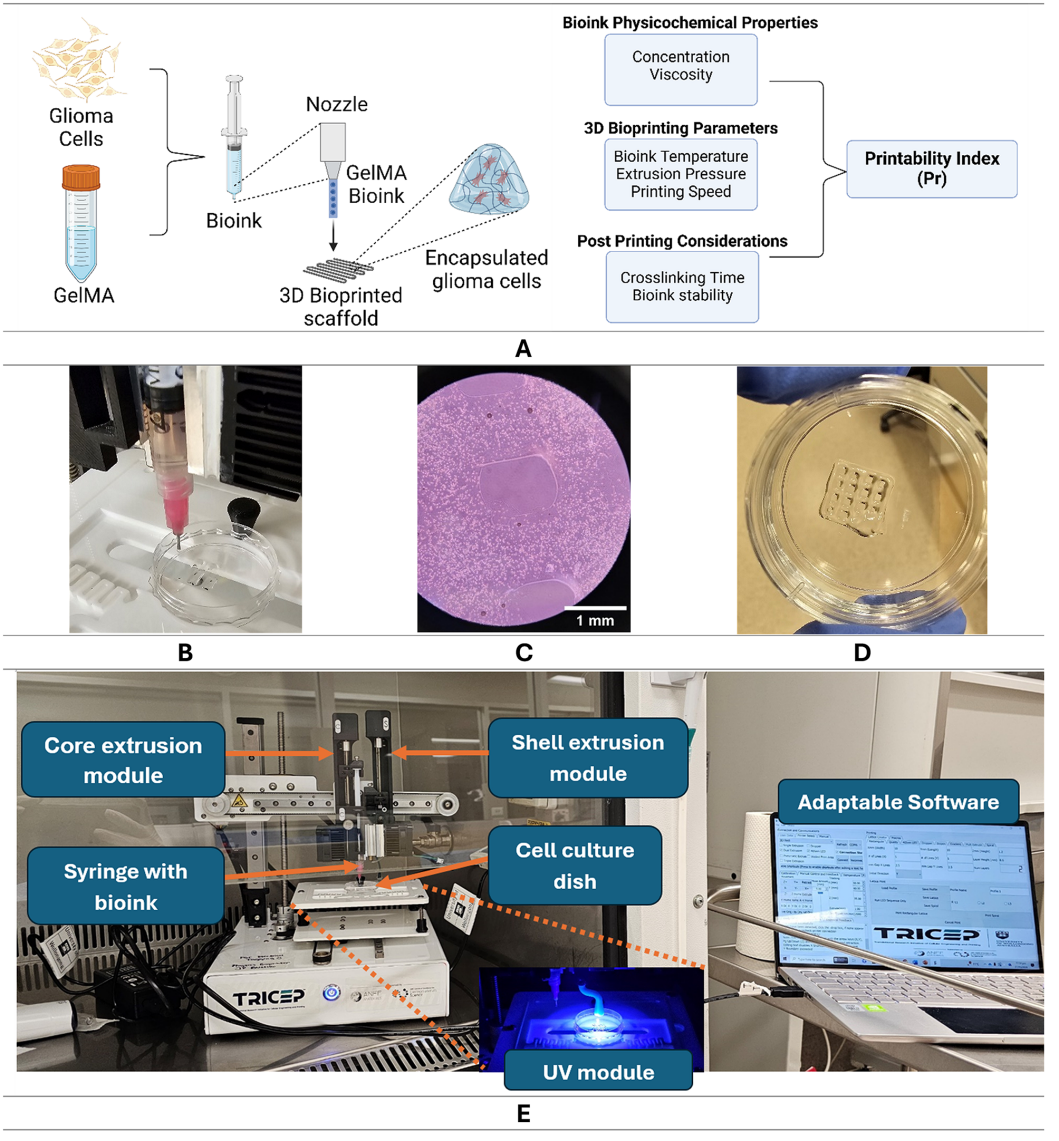
(**A**) Illustration of encapsulating glioma cells in GelMA hydrogel scaffolding and selected parameters that contribute to the printability of 3D bioprinted structure. Created in BioRender. Bustillo, J. (2025) https://BioRender.com/o77d581, (**B-D**) Pictures of the actual 3D bioprinted 8% w/v GelMA encapsulated glioma scaffolding wherein **C** shows its image under microscope (1 mm scale bar), (**E**) 3D REDI bioprinter with bioink setup inside a biosafety cabinet.

Stocks of GelMA bioinks were produced by dissolving lyophilized GelMA with Dulbecco’s Phosphate Buffered Saline (DPBS) without Calcium Chloride and Magnesium Chloride (Gibco, #14190144) resulting in different concentrations: 5%, 8%, and 10% w/v. The selected concentrations were based on published studies on the use of GelMA to model soft tissue considering its mechanical properties as it affects the diffusion and delivery of bioactive molecules^49,50^. These GelMA bioinks were stored in a laboratory freezer (−20^o^C) as stock bioinks. The frozen GelMA were thawed at room temperature before being used for cell encapsulation as shown in Fig. 1.**A**. In addition, these bioink concentrations were used to optimize the 3D bioprinter parameters, shown in Fig. 1, considering the printability of the bioink. The optimization process was done experimentally and detailed discussion on the effects of these parameters was presented in a previously published study on the use of machine learning for extrusion 3D bioprinting^51^.

Prior to bioprinting, 0.06% of Lithium phenyl-2,4,6-trimethylbenzoylphosphinate (LAP, Purity of ≥ 95% High-Performance Liquid Chromatography (HPLC), TRICEP) was added to the GelMA bioink as a photo-initiator. The recommended LAP concentration and ultraviolet (UV) exposure time were used to reduce the effect of photopolymer cross-linking to the cell viability. LAP can produce a high percentage of live cells after a UV exposure of about 1 min to 2 min, but long UV exposure can significantly affect cell viability as shown in previous published study^52^. Another published study shows that the radical formation during the activation of LAP should result in cell viability above 80% after 24 h for LAP concentrations around 0.05% and 0.067% w/v^53^. LAP has good cytocompatibility, and water solubility. It is a free radical photo-initiator used to start free radical chain polymerization in the GelMA bioink upon UV light exposure. Bioink samples were exposed to UV for about a minute to decrease the potential cytotoxic effect of free radicals to the encapsulated glioma cancer cells as suggested in literature^54^.

**Fig. 2:**
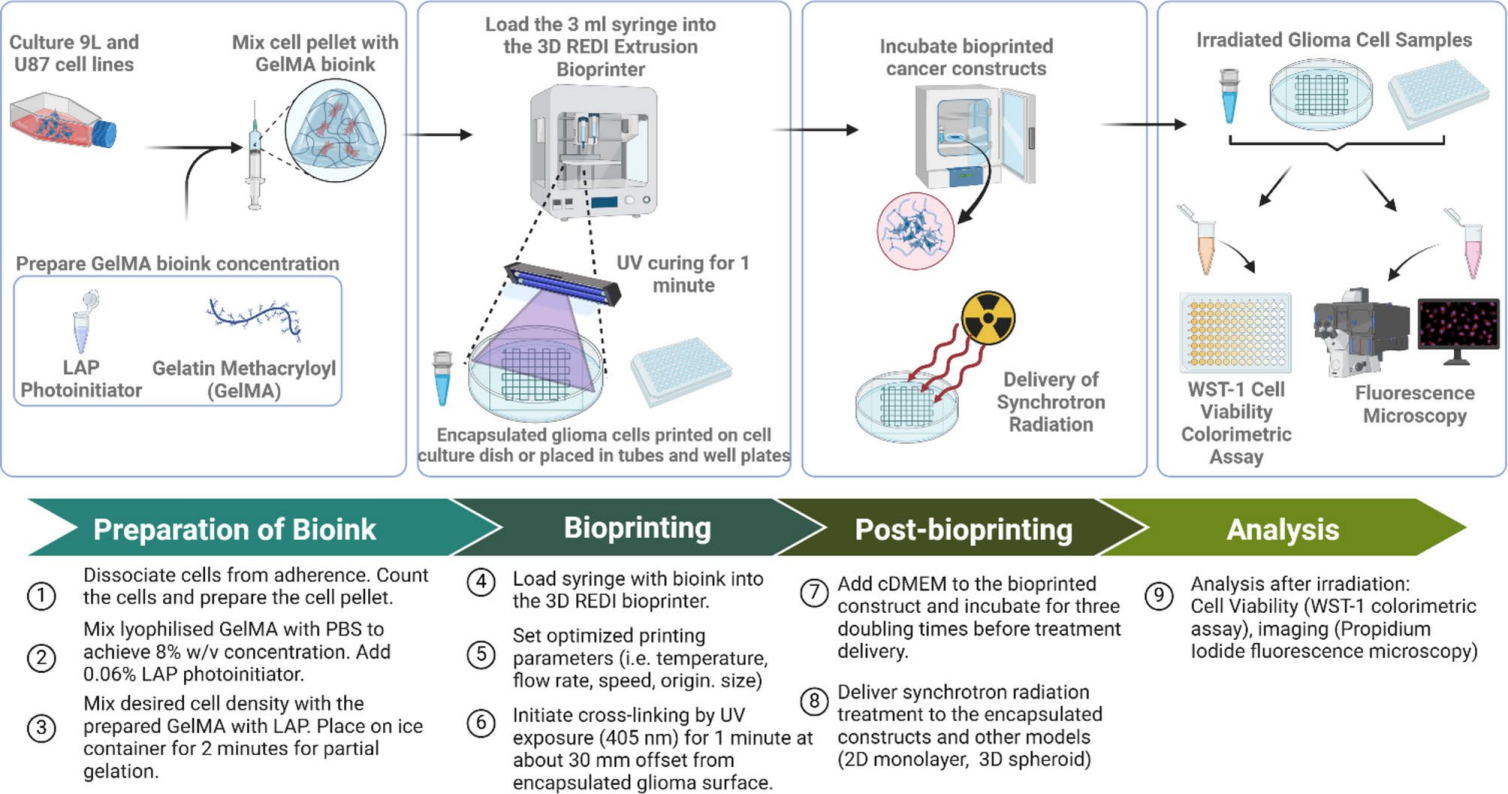
3D bioprinting protocol used in this study to create 3D in vitro models. (Created in BioRender. Bustillo, J. (2024) https://BioRender.com/b38r747)

A compact in-house 3D REDI bioprinting system (TRICEP^™^, Australia) shown in Fig. 1.**E** was used to print bioprinted lattices using the following concentrations of GelMA for optimization: 5%, 8%, and 10% w/v. This 3D bioprinting system has two mechanical extrusion units (core and shell extrusion modules) capable of extrusion and retraction of bioinks contained in 3 cc syringes. The bioink’s viscosity can be actively controlled using the Peltier thermal control units installed in the bioink syringe holder. In addition, crosslinking can be initiated using a light source module (405 nm) with a variable intensity (tricep.com.au). The bioprinter was placed inside a biosafety cabinet while being controlled by Adaptable software (TRICEP^™^, Australia). The bioprinting protocol used in fabricating a glioma biological construct is divided into four main parts as illustrated in Fig. 2: (1) preparation of bioink, (2) bioprinting, (3) post-bioprinting, and (4) analysis.

A practical way to quantify the printing quality or printability is to produce a bioprinted grid pattern and assess the shape of the printed pores of the grid using printability index (Pr) shown in Eq. 1^55^. Two layers of bioink scaffolds were printed in an orthogonal grid pattern (0^o^ and 90^o^) using a precision syringe tip of 20 Ga (Precision Tips Engineered Fluid Dispensing Nordson EFD, #7018163), and bioink strand gap of 2.5 mm.

1 $$\:Pr=\frac{\pi\:}{4}\bullet\:\frac{1}{C}=\frac{{L}^{2}}{16 A}\:\:$$

2 $$\:C=\frac{4\pi\:A}{{L}^{2}}\:$$

wherein C is the enclosed pore circularity, L is the perimeter, and A is the area of the pore. Optimal bioink and uniform strand extrusion for printing will produce the ideal square pores in the bioprinted lattice with a Pr value of 1. In contrast, poor quality bioinks will produce a watery or irregular shaped grid that will give a Pr value of < 1 (under-gelation) or > 1 (over-gelation), respectively. This printing shape index quantifies the circularity of the grid pore and its deviation from the square pore model along the x-y plane which is a straightforward way to assess the pore lattice^56^. Various combinations of bioprinting parameters such as hydrogel concentration, syringe temperature, printing speed, and bioink flow were varied to evaluate the optimal parameters that were used in producing 3D bioprinted cancer cell constructs. Five (*n* = 5) repeats per printing parameter combinations were imaged optically and analyzed using imaging software.

### Bioprinting with glioma cancer cells protocol

#### Glioma cell culture

9L gliosarcoma (9L), a Fischer rat gliosarcoma, was acquired from the European Collection of Cell Cultures (ECACC). This cell line is composed of sarcoma and glioblastoma appearance which is useful for rat glioma tumor model. Another cell line used is U87 malignant glioma (U87). This is a human primary glioblastoma, and astrocytoma cell line commonly utilized in neuroscience and cancer research. Both cell lines were cultured in T75 flasks with Gibco® clear complete Dulbecco’s modified eagle medium (c-DMEM Gibco, #21063-029) with 4.5 g/L D-Glucose, L-Glutamine, 25 mM HEPES, 1% penicillin and streptomycin and 10% fetal bovine serum (FBS) following the protocol of previous study^10^. Incubation conditions for cell culture were 37 ^o^C and 5% (v/v) CO_2_. The cells were passaged into different flasks with c-DMEM before delivering the radiation beams.

#### Encapsulation of glioma cells using 8% w/v GelMA bioink

Figure 2 illustrates the 3D bioprinting protocol used in this study. Adherent glioma cells in a T75 cell culture flask were dissociated via Trypsin. It was counted using a hemocytometer and centrifuged using the setting 1500 rpm for 5 min. The GelMA hydrogel stock (8% w/v GelMA), and cell pellet of malignant glioma cell culture (9L and U87, separately prepared) were mixed into a 3 ml Luer lock syringe (Hapool, SS03LL) through repetitive pipetting. The cells were precisely combined with GelMA to avoid clumping of cells. It was then put on ice for about 2 min to initiate gelation. Lastly, the syringe was covered with a precision syringe tip of 20 Ga or about 0.603 mm inner diameter (Precision Tips Engineered Fluid Dispensing Nordson EFD, #7018163). 3D GelMA encapsulated glioma was prepared with the cell density of 120,000 cells/ml and 100,000 cells/ml for 9L and U87 cell lines, respectively. These cell densities are based on the 3D spheroid optimization done for each cell line considering the spheroid diameter, contraction time, and growth^57^. 3D encapsulated glioma blobs were placed in 96-well plates (Corning Incorporated costar®, Ref 3599) and PCR tubes to prepare the in vitro irradiation setup. Clear c-DMEM was added to all glioma blobs and returned inside the incubator.

#### 2D monolayer and 3D spheroid glioma cells

Two-dimensional (2D) monolayers of both 9L and U87 cells were prepared in 96-well plates using the same cell density applied in 3D cell encapsulation previously discussed. In addition, 3D spheroids of these cell lines were generated by seeding 96 well, with lid and round bottom, ultra-low attachment (ULA) polystyrene plates (Corning®, 7007) following the cell density formerly used with a working volume of 200 µl. ULA plates with cells were centrifugated at 1500 rpm for 5 min. Then, the ULA plates were placed in the incubator to allow cell contraction and growth to form the spheroids for about three doubling times (or about 3 days). The generated spheroids were recovered by pipetting for irradiation. Monitoring of spheroid growth was done by measuring the diameter of the glioma cell aggregate over time using the brightfield mode of Leica fluorescence microscope (Leica Microscope Systems).

#### 3D bioprinting of glioma lattices

Syringe with bioink and cells were loaded in the 3D REDI bioprinter. The printing temperature (^o^C), speed (mm/min), and flow (ml/mm) were adjusted using the Adaptable software depending on the concentration of the GelMA bioink. Photocrosslinking was done using a built-in 405 nm light-emitting diode (LED) with 100% intensity (about 1.5 mW/cm^2^) setting and a light source to bioprinted construct distance of about 30 mm. Glioma cancer lattice constructs ($\:10\times\:10\times\:1.2\:{mm}^{3}$) were printed in 35 × 10 mm tissue culture dish with a 9.40 cm^2^ growth area (SPL Life Sciences, 11035) using optimized bioprinting parameters as shown in Fig. 1.**D**. Bioprinted lattice structure closely simulates the porous structure of soft tissue that enables good migration of cells and diffusion of nutrients. This improves the ability of cells to properly grow within the bioink scaffolding. In addition, this structure pattern is advantageous to have good vascularization when implanted in a body^58,59^.

The 3D REDI extrusion-based bioprinter was utilized in fabricating the bioprinted constructs. GelMA solution was loaded into a 3 mL Luer lock syringe and maintained to a set syringe temperature of about 20^o^C to improve bioink’s gelation. The bioprinted construct was immediately exposed to a 405 nm LED source after printing to initiate crosslinking. A separate set of cell-laden bioinks were prepared by mixing 8% GelMA with a cell density of $\:1\times\:{10}^{6}$ cells/ml for lattice bioprinting.

### Radiological characterization and synchrotron radiation dosimetry

**Fig. 3:**
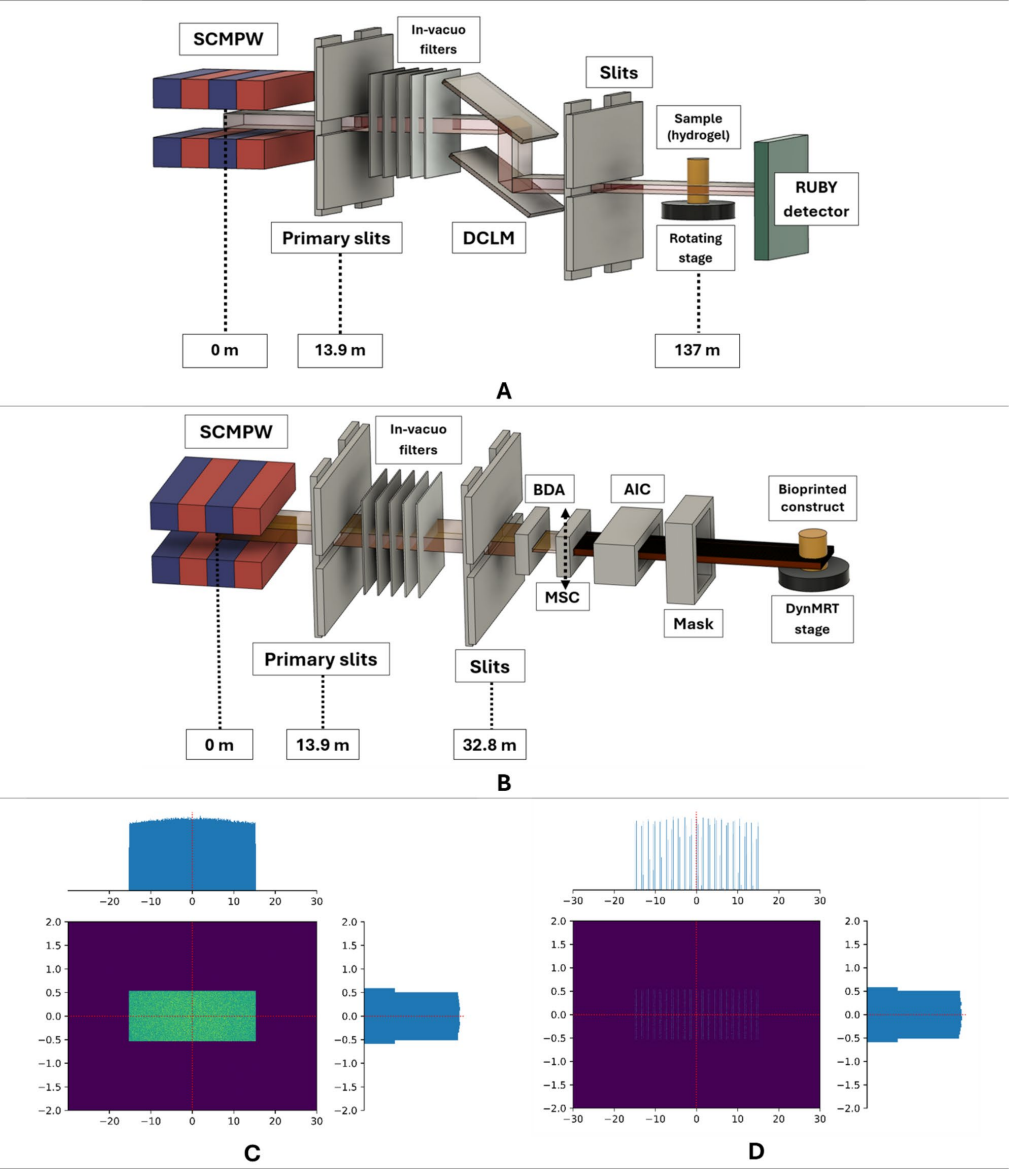
Simplified illustration (not to scale) of the experimental setup at the Australian Synchrotron- Imaging and Medical Beamline with approximated distances relative to the SCMPW radiation source: (**A**) hutch 3B for the synchrotron computed tomography imaging in attenuation coefficient measurement, (**B**) hutch 2B in delivering synchrotron broad-beam (SBB) and microbeam radiation therapy (MRT), (**C** & **D**) Geant4 Monte Carlo simulated SBB and MRT beam relative dose profiles in arbitrary units (a.u.), respectively. Superconducting multipole wiggler (SCMPW), dual-crystal Laue monochromator (DCLM), beam defining aperture (BDA), removable multislit collimator (MSC), air ionization chamber (AIC).

Experimental monochromatic attenuation coefficients of 10% w/v GelMA and 30% w/v Lutrol® F-127 Pluronic were measured at the hutch 3B of the Imaging and Medical Beamline (IMBL) of the Australian Synchrotron. This is to characterize the radiological property of these two hydrogel polymers used in tissue engineering and 3D bioprinting. Lutrol was only used in calibrating the machine, while GelMA was used in preparing bioink for 3D bioprinting experiments. Note that these percentages of hydrogel and DPBS mixture were chosen as these are used in calibrating the 3D REDI bioprinter before optimizing the bioprinting parameters. The setup of the instrumentation is illustrated in Fig. 3. This beamline hutch is about 140 m from the source to produce wider synchrotron radiation beam and to have a phase-contrast imaging capability by increasing the sample-to-detector distance^60^. The following synchrotron micro- CT imaging parameters were used: 3 Tesla magnetic field of superconducting multipole wiggler (SCMPW), synchrotron radiation beam filters composed of carbon (C) and aluminium (Al) (C(0.45), C[hd](5), C[hd](10), Al(1)-Al(1), Al(1)-Al(1)), and a RUBY detector (Monash University, Dynamic Imaging laboratory) with pixel resolution size of 17.5 μm and about 45 mm horizontal field of view^25,61^. This imaging detector has a converter screen scintillator (Gd_2_O_2_S: Tb) and a scientific complementary metal-oxide semiconductor (CMOS) sensor. Technical details of the beamline including the instrumentation distances can be found in published studies^60,62^. Monochromatic synchrotron radiation beams of 8 different energies (30 keV ≤ E ≤ 65 keV) were delivered in 5 keV increment by adjusting the rocking angle ∆θ of the dual-crystal Laue monochromator (DCLM) crystal and measuring the radiation flux using the beamline ionization chamber. Details of the acquisition of projection data, image reconstruction and corrections are discussed in previously published studies^25,26^. The process of selecting the wavelength of the synchrotron radiation using DCLM can be expressed mathematically using the Bragg equation shown in Eq. 3. The linear attenuation coefficient (µ) can be derived from the reconstructed CT images using inverse Radon transform $\:\left({\mathcal{R}}^{-1}\right)$ following the expression of output intensity $\:I\left(x,\varphi\:,\lambda\:\right)$ shown in Eq. 4^62,63^.

3 $$\:n\lambda\:=2d\text{sin}\left(\theta\:\right)$$

where $\:\theta\:$ is the angle between the silicon crystal plane with distance d between crystal planes, n is a whole number, and $\:\lambda\:$ is the wavelength

4 $$\:I\left(x,\varphi\:,\lambda\:\right)={I}_{o}\text{exp}\left(-{\int\:}_{L\left(x,\varphi\:\right)}^{}\mu\:\left(x,\varphi\:,\:t,\:\lambda\:\right)dt\right)={I}_{o}\text{exp}\left(-\mathcal{R}\mu\:\left(x,\varphi\:,\lambda\:\right)\right)\:$$

where $\:{I}_{o}$is the incident intensity, $\:L\left(x,\varphi\:\right)$ is a line with a path length t, $\:\varphi\:$ is the rotation angle, x is a point in the projection plane, and $\:\mathcal{R}$ is the Radon transform

In addition, a Siemens Inveon^™^ PET/CT preclinical imaging machine (Siemens Medical Solutions) was used in acquiring CT images of the hydrogels using polychromatic kVp energies. The recommended CT acquisition parameters were used to acquire projection images: 112.94 μm effective pixel size, 0.11 mm slice thickness, and a Feldkamp filtered kernel. The mean CT number in Hounsfield Units was measured for all the reconstructed CT images.

The irradiation experiment was done at the experimental hutch 2B of the IMBL of the Australian synchrotron using dynamic delivery. The Australian synchrotron is a third-generation synchrotron light source working on “top-up” mode. This is to maintain a constant 200 mA of beam current within the synchrotron storage ring. Various optics and beam controlling components (i.e. slits, in-vacuo filters, beam defining aperture) are shown in Fig. 3. The synchrotron X-ray radiation was produced using a 3 Tesla superconducting multipole wiggler (SCMPW). Two synchrotron beam modes were utilized: (a) SBB, and (b) MRT. Table 1 shows the summary of the synchrotron beam spectrum characteristics and beamline setup.

MRT is a preclinical synchrotron radiotherapy modality that delivers an array of micro sized beams to treat radio-resistant cancers such as brain tumors. This is done by placing a tungsten MSC to produce spatially fractionated beams as illustrated in Fig. 3.**D**. The synchrotron radiation beam will produce an SBB, illustrated in Fig. 3.**C.**, if the MSC is not inserted into the radiation path. In this study, both synchrotron beam modes were delivered to biological samples comparing their relative response. Note that the Monte Carlo simulated data shown in Fig. 3 are relative dose profiles in arbitrary units for illustration purposes.

**Table 1.**
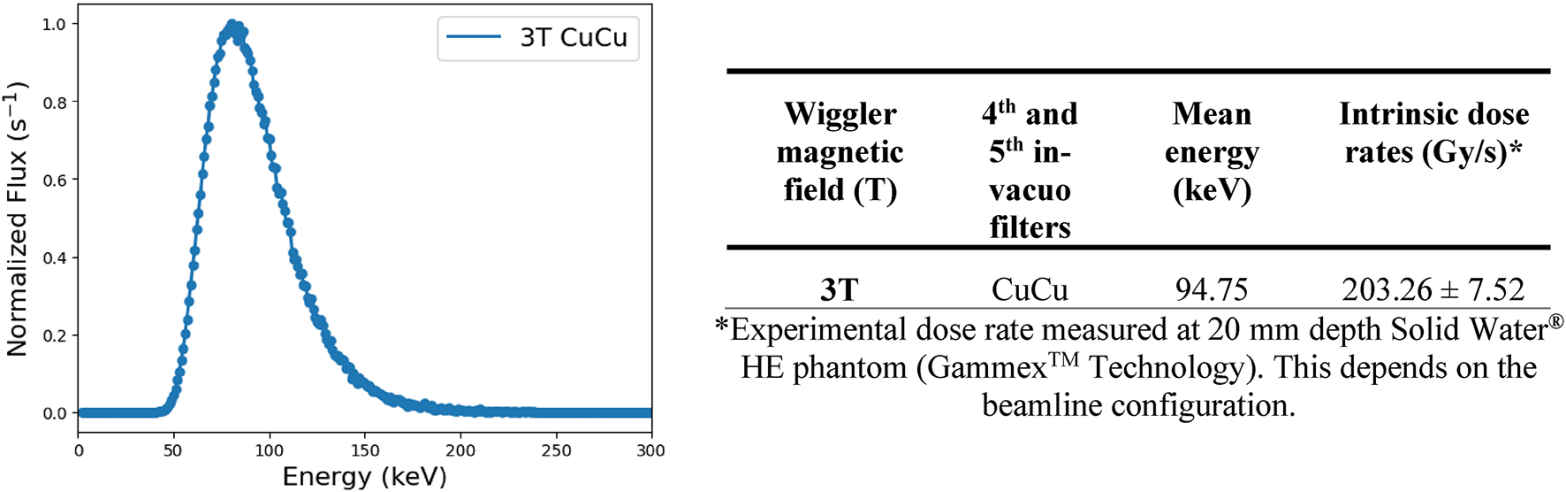
Geant4 Monte Carlo simulated synchrotron radiation spectrum and setup details used for the in vitro irradiation experiment at the IMBL-AS. *Experimental dose rate measured at 20 mm depth solid Water® HE phantom (GammexTM Technology). This depends on the beamline configuration.

Established dosimetric protocols of the beamline for both SBB and MRT were done using a $\:10\times\:10\times\:10\:{cm}^{3}$ Solid Water® HE (Gammex™ Technology) and a calibrated PinPoint ionization chamber (IC) (PTW 31022, Freiburg, Germany). The following reference conditions were utilized: $\:20\times\:20\:{mm}^{2}$ SBB field size at 20 mm depth of the phantom slabs. Then, a silicon strip diode detector (SSD) fabricated on a p-type 50 μm thick epitaxial substrate (EPI) and an X-Tream dosimetry system with micron-scale spatial resolution developed by the Centre for Medical Radiation Physics- University of Wollongong Australia were calibrated using the same reference condition and were used to measure the microbeam peak and valley doses across the phantom. This detector system is suitable for very high dose rate and gradient present within the micron striated treatment beams. It has a large dynamic range that can resolve both the peak and the valley doses^64^. Technical details of the radiation detector system are available in a published study^65^. Prior to each irradiation, EBT3 films (Ashland, Bridgewater, NJ, USA) were placed on the front and back side of the 3D bioprinted sample to verify the delivered radiation geometry. Films were also used to verify the radiation geometry downstream.

One relevant relative quantity in delivering spatially fractionated MRT is the peak-to-valley dose ratio (PVDR) shown in Eq. 5. This is dependent on the X-ray beam energy, field size and the center-to-center distance of the MRT beams. The sparing effect on the healthy tissue depends on the valley dose as it approaches the broad beam dose tolerance^11,14^.

5 $$\:PVDR=\frac{{Dose}_{Peak}}{{Dose}_{Valley}}\:$$

**Fig. 4:**
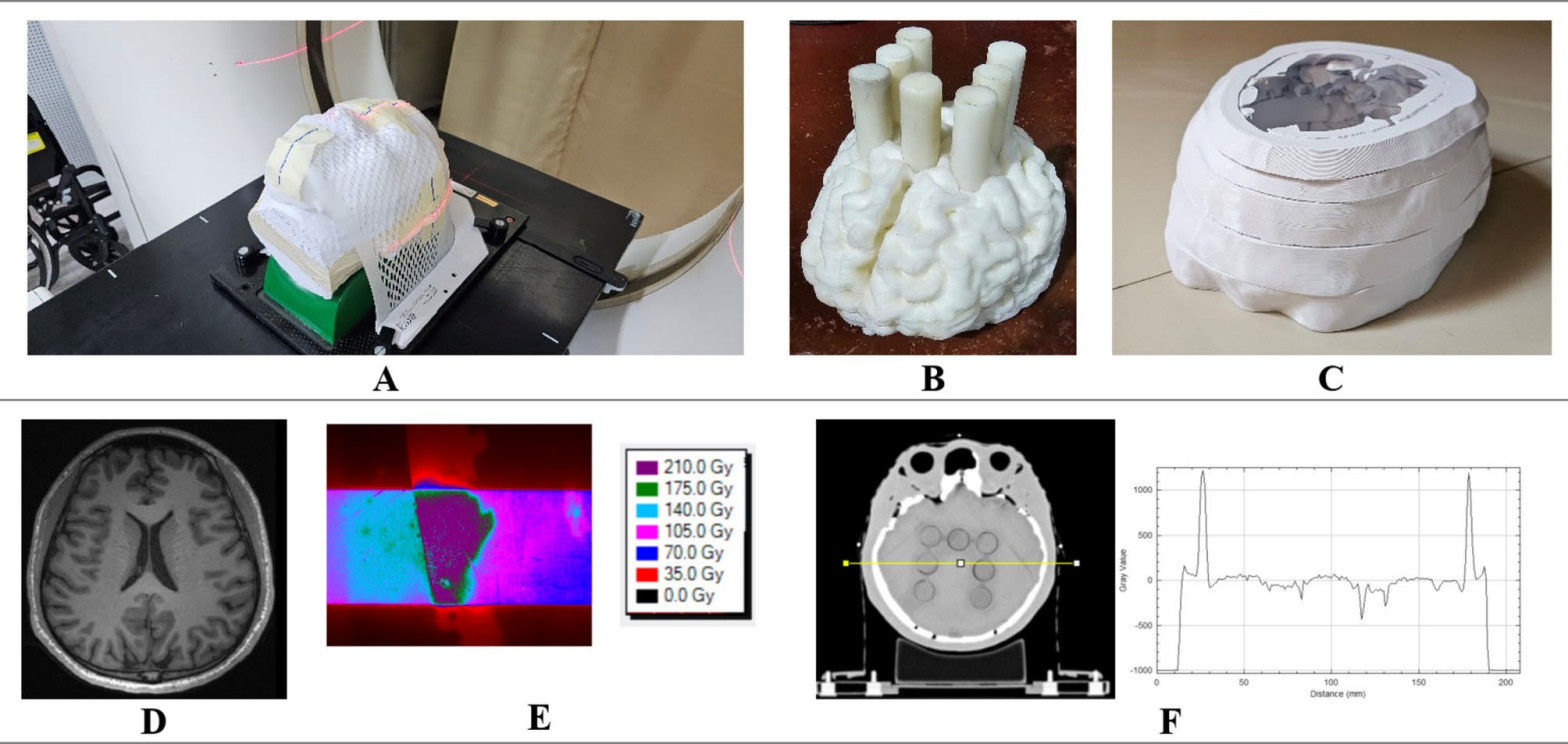
(**A**) 3D printed head phantom in a CT simulation machine for CT number characterization, (**B**) 3D printed adult brain with cylindrical inserts, (**C**) Selected external layer of the 3D printed head phantom, (**D**) Actual MRI brain slice of a human head, (**E**) radiochromic film 2D SBB dose map inside the 3D printed head phantom showing the skull and brain, (**F**) CT image and profile of the 3D printed head phantom.

3D printed phantoms were utilized by incorporating encapsulated glioma cells inside a rat and an adult head phantom. The design and fabrication details of the rat phantom made from polylactic acid plus filament (PLA+, eSun^™^, China) were discussed in a previous study wherein it was shown to be soft tissue equivalent for the synchrotron radiation energies^26^. On the other hand, the adult head phantom is composed of PLA+, gypsum plaster (Uni-PRO, Australia), and nylon (Ultimaker, The Netherlands) to mimic the soft tissue, skull, and brain, respectively. The protocol in 3D printing of a head phantom using Ultimaker S5 3D printer (Ultimaker, The Netherlands) was based in a previous study^66^. Figure 4 shows the 3D printed adult head phantom based on a head magnetic resonance imaging (MRI) image set acquired using a 3T Prisma MRI system (Siemens Medical Solutions). The fabricated phantom was imaged in Siemens Emotion 16-slice CT machine (Siemens Medical Solutions) using 80, 110, and 130 kVp, H31s convolution kernel, and 1 mm slice thickness following the clinical imaging protocol of the facility for radiotherapy CT simulation of an adult head. The 3D printed head phantom was divided into axial slices wherein the cerebrum part at the level of the lateral ventricles was used in irradiating the glioma in vitro samples. Cylindrical detector inserts were designed and fabricated that can fit inside the head phantom.

**Fig. 5:**
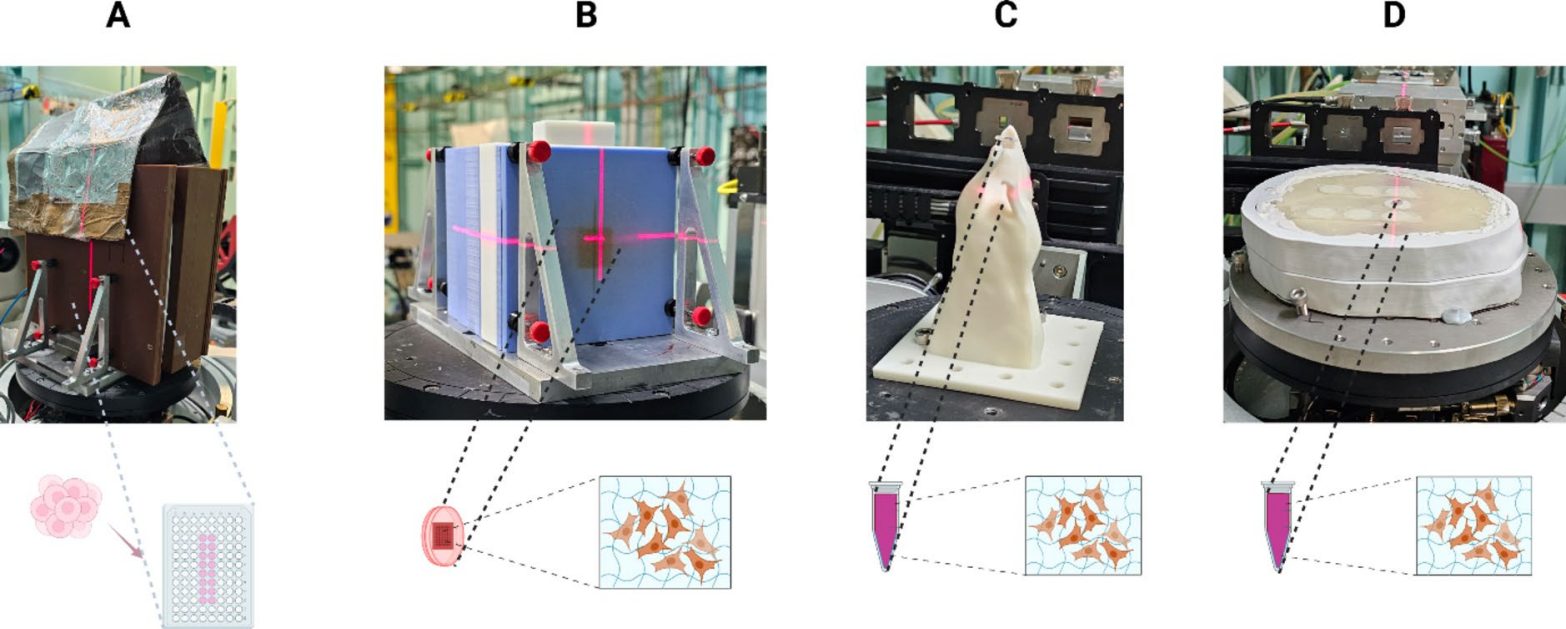
Different irradiation setup used to deliver synchrotron broad-beam and microbeam radiation beams to the in vitro samples: (**A**) Combination of RMI457 slabs and PLA + slab insert for 96-well plate (Lead sheet was used to localize the beam during column irradiation.), (**B**) 3D printed PLA + slab insert for cell culture dish together with Solid Water® HE, (**C**) 3D printed rat phantom, and (**D**) 3D printed adult anthropomorphic head phantom. Created in BioRender. Bustillo, J. (2025) https://BioRender.com/f96g579.

### In vitro experiment protocol

The IMBL hutch 2B was used to deliver both SBB and MRT as described in Table 1. Three in vitro experimental setups were used in this study: (1) single column irradiation of 96-well plates, (2) dynamic scan irradiation of cell culture dishes, and (3) 3D GelMA encapsulated glioma in PCR tubes incorporated inside a 3D printed rat and a 3D printed human head phantom as shown in Fig. 5. Prior to each in vitro irradiation, radiation doses were measured at the same depth where the cell samples were placed. After irradiation, the in vitro cell cultures were returned to the incubator and monitored daily. Culture media were changed as needed.

#### Bioprinted cell viability analysis and fluorescence imaging

All the cells were kept in the cell incubator for three doubling times before doing post experiment analysis illustrated in Fig. 2. Cell viability was assessed using a Cell Proliferation Reagent WST-1 (Roche Diagnostics, #05015944001). The WST-1 cell viability assay is a colorimetric assay based on the cleavage of tetrazolium salt WST-1 or 4-[3-(4-Iodophenyl)−2-(4-nitrophenyl)−2 H-5-tetrazolio]−1,3-benzene Disulfonate (light red) to produce formazan dye (dark red) through cellular mitochondrial dehydrogenases by metabolically active cells. The amount of formazan dye corresponds to the viable cells’ metabolic activity. A spectrometer-based absorbance microplate reader (SPECTROstar® Nano, BMG Labtech) was used to check the absorbance of in vitro cell culture. Absorbance measurements were acquired and compared to the cell viability of the control sample (0 Gy). Cell viability assays done for bioprinted GelMA in published studies have shown the efficacy of using a colorimetric assay technique such as WST-1 to quantify the viable cells after giving a treatment^67^. This is an important test to understand the differences in the response of a 2D monolayer cells to a 3D bioprinted cells encapsulated inside a biopolymer.

Propidium iodide (PI, Sigma Aldrich Ltd.) fluorescence imaging was added on both 2D, and 3D cell cultures at a concentration of 1 µl/well to the culture medium to image the cells with compromised membrane. PI is a fluorescent intercalating agent used to stain cells and nucleic acid. This is also utilized in identifying dead cells because it is not permeant to live cells. Leica DMC2900 fluorescence microscope (Leica Microscope Systems) was used to acquire multiple images in mosaics following the same optimized imaging parameters. Image analysis was done using Leica LAS X Application (Leica Microscope System), and Fiji ImageJ software (National Institute of Health, USA) by adjusting the low threshold of the image and measuring integrated density of the fluorescence signal.

### Statistical analysis

Data presented in this study were all presented in mean, standard deviation, and standard error of the mean. Further analysis was done in Microsoft Excel spreadsheet using two sample unequal variance two-tailed t-test. Difference was reported statistically significant if *p* < 0.05 (*) as presented in the figure. Consistency of the experimental procedure was verified by using two different cell lines in this study.

**Fig. 6:**
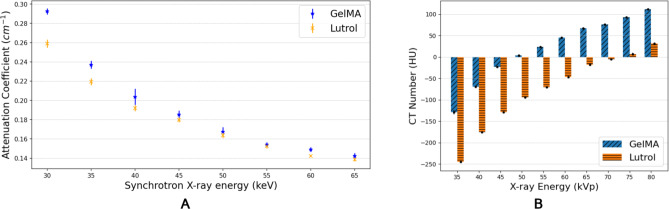
(**A**) Experimental monochromatic attenuation coefficient based on synchrotron CT imaging, and (**B**) CT number measurement of hydrogel samples using Siemens Inveon CT machine. Error bars represent the standard deviation. (*n* = 5 repeats).

## Results

### Hydrogel attenuation coefficient and CT number

The experimental monochromatic attenuation coefficients and mean CT numbers of GelMA and Lutrol are shown in Fig. 6. The attenuation values of GelMA and Lutrol are within ± 5% similarity from 45 keV up to 65 keV. Large difference was observed at lower photon energies because of the high dependence of the X-ray interaction on the sample’s effective atomic number^25,68^. The lower attenuation values are due to the presence of pores in the hydrogel mixture which is important for the cells to have access to culture media. This is consistent with the low mean CT number measurement shown in Fig. 6.**B** specially at lower energies (35 to 50 kVps). The ratio of the PBS and hydrogel was based on the supplier’s recommendation. GelMA and Lutrol were observed to have a CT number comparable to water for the energies 50 and 75 kVp, respectively. The measured CT numbers above the previously mentioned energies are all within the range of soft tissue.

### Microbeam radiation therapy dosimetry

Dosimetry protocol for SBB and MRT at the IMBL established in previous publications were followed by measuring first the SBB followed by MRT beam characterization which involves measurement of peak and valley doses^10,26,64^. Prior to every cell irradiation experiment, treatment speeds were determined by measuring the SBB and MRT valley doses at the depth of sample location within the phantom based on the synchrotron beam parameters shown in Table 2. The PVDR is shown to be the highest for the rat phantom. This is due to less scattered radiation present in the small rat phantom producing lower valley dose. Thus, more prominent peaks were observed. On the other hand, the heterogeneous human head phantom produced the lowest PVDR because of its large size and presence of heterogeneity which generated more scattered radiation contributing to valley dose.

**Table 2:** Beam setup for synchrotron broad-beam and microbeam radiation therapy used in this study. All these quantities were measured at the in vitro model sample position. Uncertainties were evaluated within one standard deviation.

		Rectangular slab phantom	Rat phantom	Human head phantom
SBB	Intrinsic dose rate (Gy/s)	203.26 ± 7.52 at 20 mm	214.04 ± 1.74 at 6.25 mm	56.68 ± 3.76 at 20 mm
	Beam size & BDA	20 mm × 20 mm; 0.546 mm	10 mm × 10 mm; 0.25 mm	10 mm × 10 mm; 0.546 mm
MRT	Beam size & BDA	20 mm × 20 mm; 1.0 mm	10 mm × 10 mm; 1.0 mm	10 mm × 10 mm; 0.546 mm
	PVDR	**13.51 ± 3.77**	**50.78 ± 2.54**	**9.95 ± 0.76**

SBB: Synchrotron Broad-Beam, MRT: Microbeam Radiation Therapy, BDA: Beam Defining Aperture, PVDR: Peak Valley Dose Ratio.

**Fig. 7:**
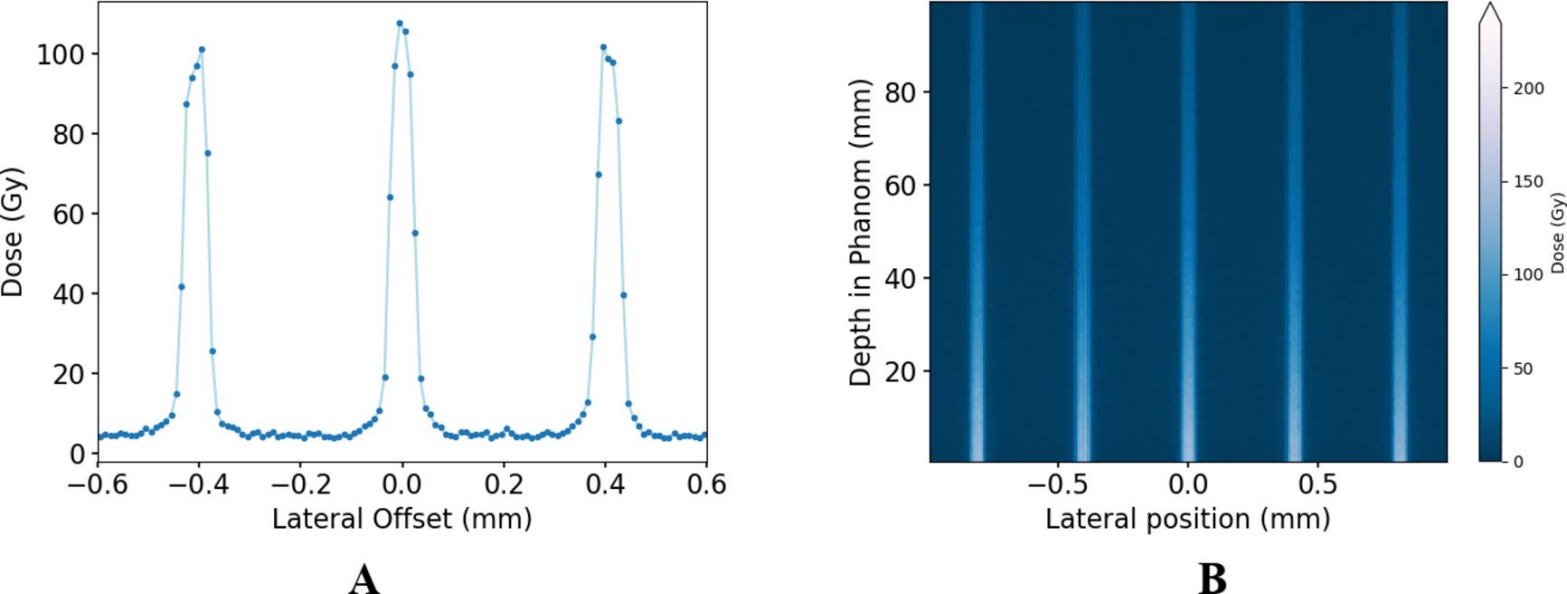
(**A**) Geant4 Monte Carlo calculated microbeam dose profile at 20 mm depth, and (**B**) Two-dimensional percent depth dose in a Solid Water HE Phantom. Peak and valley doses were verified experimentally for all the phantoms used.

Figure 7shows the calculated MRT lateral dose profile and 2D percent depth dose inside a phantom based on a validated Geant4 Monte Carlo simulation of the synchrotron beamline following the experimental parameters^69^. This is done to verify experimental measurements such as the acquired PVDR and dose profile before in vitro irradiation.

### Bioink optimization

The optimal concentration of GelMA has been reported to be around 5–10% w/v for 3D bioprinting and other cell related research. The GelMA concentration is an important factor to consider in 3D bioprinting as the cell viability decreases with increasing concentration due to its rigidity and low porosity^40^. The increase in GelMA’s stiffness is due to the increase in covalent bonds as the concentration increases. The Young’s modulus (measure of compressive stiffness) of 5–10% GelMA concentration is around 3.08 KPa and 34.9 kPa^70^. In addition, hydrogel encapsulation can cause stress to the cells that will reduce its cell viability. Although GelMA is commonly printed using 10–30% concentrations, it is suggested by published studies to use 7 to 15% GelMA concentration to have homogeneous printing and to maintain its shape considering the hydrogel’s viscosity and cytocompatibility^43,71^. Thus, this study used 5%, 8%, and 10% w/v GelMA concentrations in optimizing bioprinting parameters. Table 3 shows the optimized 3D bioprinting parameters and images of the 3D bioprinted lattice for each investigated GelMA concentration. It is not recommended to use less than 5% of GelMA due to its watery appearance that will not produce a lattice structure as reported by previous study^43^. On the other hand, greater than 10% concentration will produce stiffer lattice that can affect cell viability, and proliferation^43,44^. The printability index shown in Eq. 1 was used to assess the geometrical accuracy of the 3D bioprinted lattice.

**Table 3.**
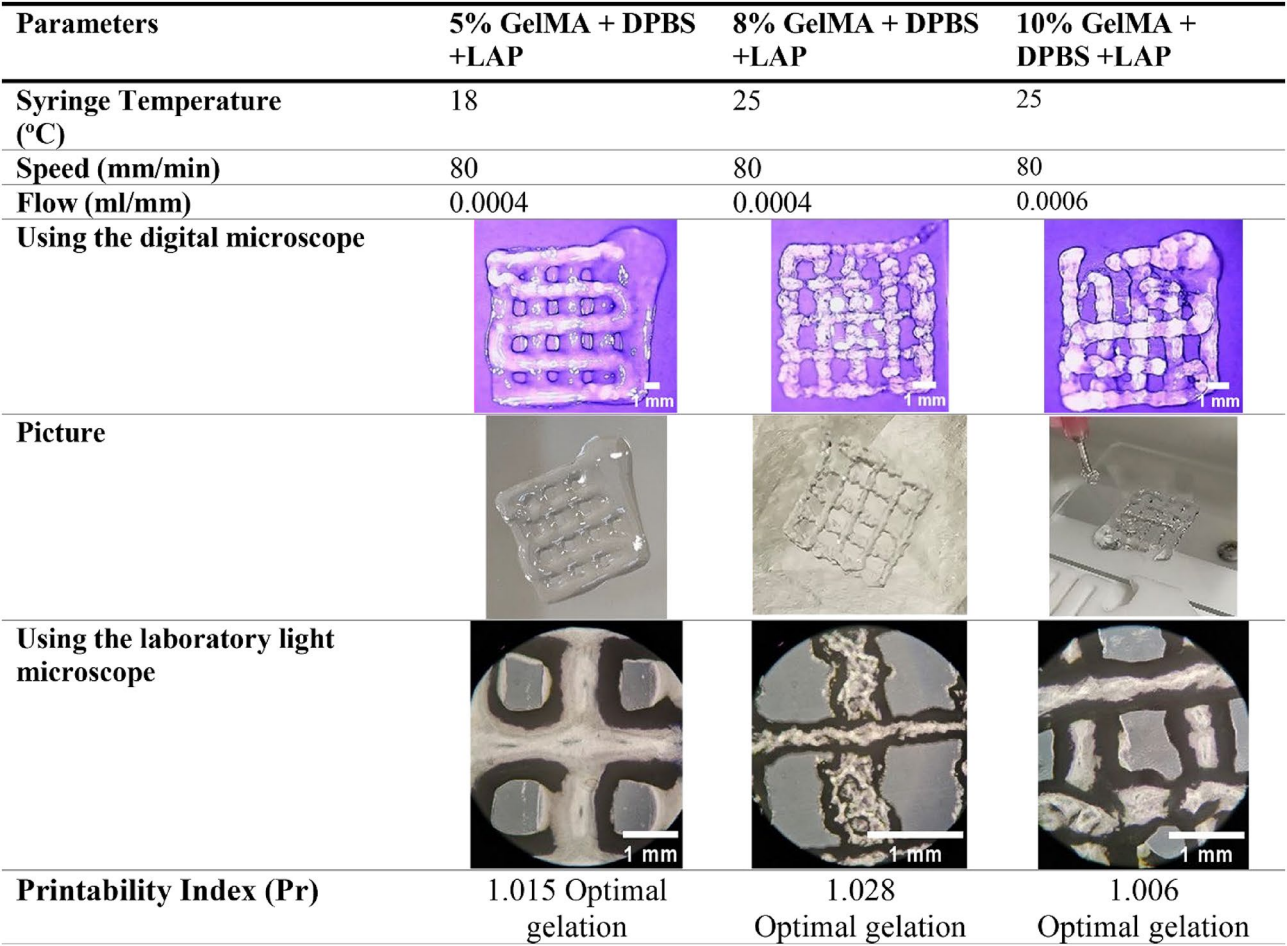
Optimized GelMA concentrations and 3D bioprinting parameters assessed in this study.

### Comparison of glioma cell monolayer, spheroid, and 3D GelMA construct

**Fig. 8:**
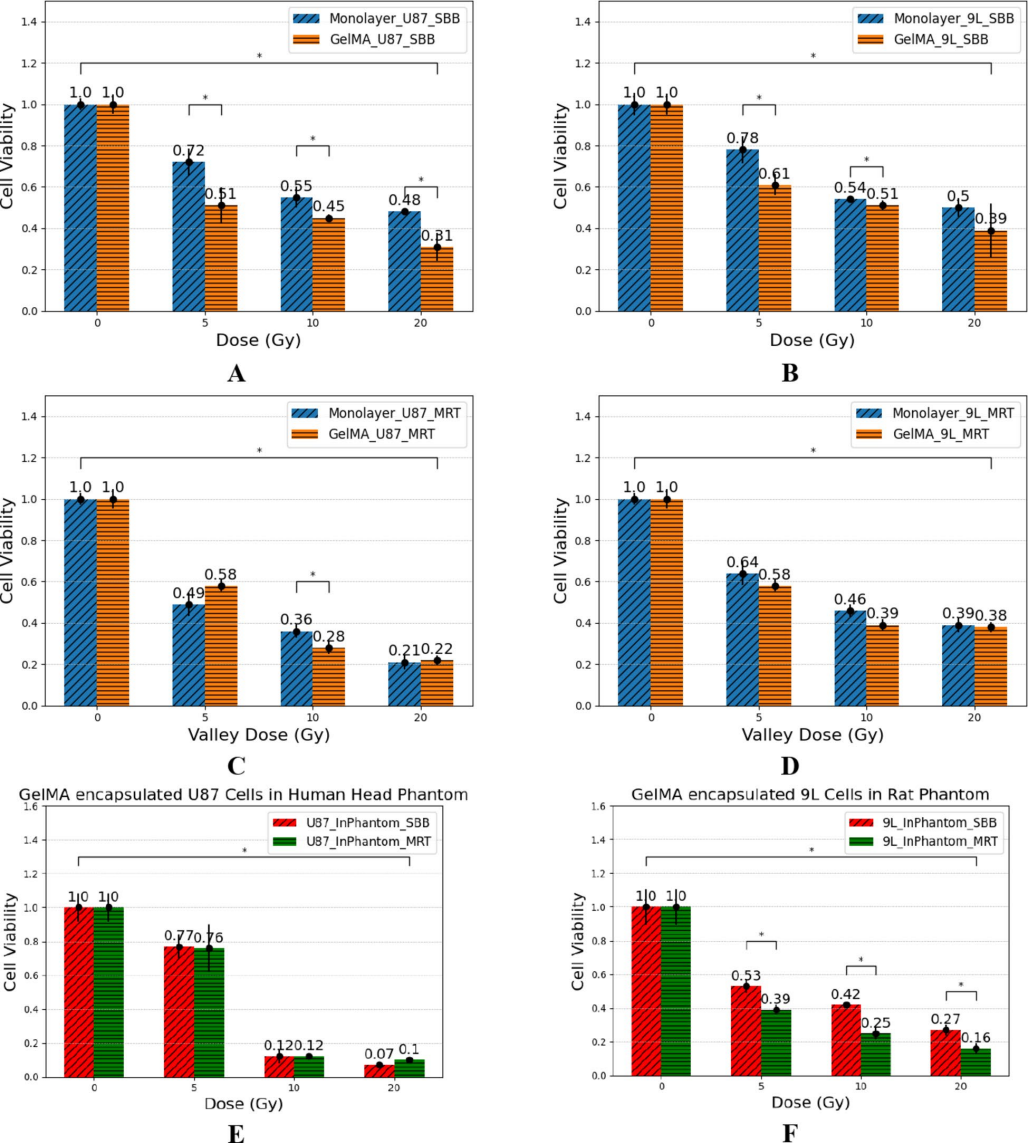
U87 (**A**,** C**,** E**) and 9L (**B**,** D**,** F**) cell line normalized cell viability for both synchrotron broad-beam (SBB) and microbeam radiation therapy (MRT) delivered to 2D monolayer cell model (blue) and 3D GelMA cell model (orange). Cell viability assay was done 72 h. after irradiation. Four replicates were done per treatment dose. Error bars represent standard error of the mean and asterisk * represents *p* < 0.05. Note that the percentages were normalized wherein 1 is equivalent to 100% cell viability.

Figure 8 shows the cell viability of both U87 and 9L cell monolayers and 3D GelMA encapsulated cells is decreasing as the delivered dose increases for both SBB and MRT. Significant differences can be seen between monolayer and 3D GelMA for all the delivered SBB doses, except for 9L 20 Gy due to large error bars. On the other hand, only the U87 10 Gy valley dose of MRT was observed to have a significant difference between cell monolayer and 3D GelMA. The observed difference between the cell viability of monolayer and 3D GelMA cells can be attributed to the uniform dose delivery in SBB which causes more uniform damage to the irradiated cancer cell models. This warrants more experimental investigation in future studies to understand the effect of synchrotron radiation dose delivery to different cancer models.

#### WST-1 cell viability assay

Generally, delivering MRT valley doses kills more U87 and 9L cells compared to SBB as shown in its lower cell viability (Fig. 8C & **D**). This can be explained by the combination of high microbeam peak doses that may cause necrotic cell death and the lower microbeam valley dose causing bystander effects to cells not directly exposed to radiation. Cell viability assay was done 72 h. after irradiation to observe the biological effect of both peak and valley doses as done in previous study^72^. Furthermore, the response of 9L cells to MRT has been shown to produce more cell death than SBB in clonogenic cell survival against the valley dose due to the presence of high peak dose^10^.

Figure 8E & **F** shows the cell viability of 3D GelMA glioma cells placed inside a 3D printed human head phantom and rat phantom. Both U87 and 9L cell lines show to have a decrease in cell viability as the SBB and MRT valley dose increases. It is worth noting that the 9L encapsulated cells placed in a rat phantom show to have significant difference between the cell viability of SBB and MRT for all the doses delivered. This is attributed to the high PVDR measured inside rat head phantom as shown in Table 2. The radiological tissue equivalence characterization of the 3D printed rat phantom is presented in a previous study^26^. High peak dose present in the rat phantom produces more cell killing which is reflected in the decrease of cell viability after MRT irradiation. On the other hand, U87 inside the 3D printed head phantom did not show significant differences between SBB and MRT’s cell viability due to the lower measured PVDR. The human head phantom has three heterogeneities which include brain, soft tissue, and bone with experimental average CT number (Siemens Emotion 130 kVp, 88 mA) of 8.46 ± 4.85, 99.68 ± 22.26, and 932.24 ± 89.83 HUs, respectively. Its heterogeneity (presence of bone, and brain materials) and sample depth (approx. 20 mm) affect the effectiveness of spatially fractionating synchrotron beams using MRT.

**Fig. 9:**
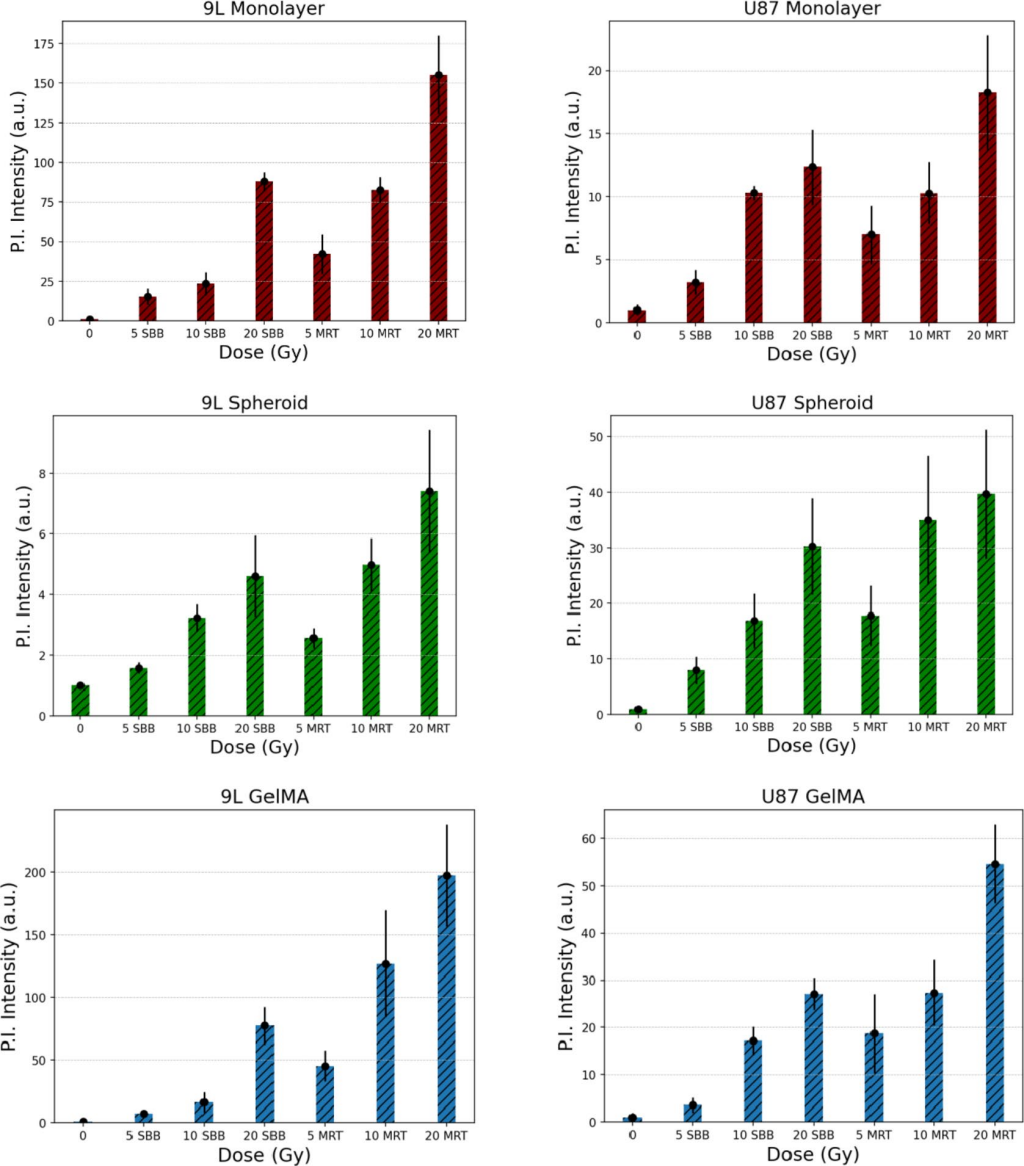
Propidium iodide average fluorescence intensity ± standard deviation of the mean (in arbitrary units, a.u.) normalized to the control (0 Gy) for 2D monolayer, 3D spheroid, and 3D GelMA encapsulated cells measured three days after treatment delivery. (*n* = 5).

#### Propidium iodide fluorescence imaging

Figure 9shows that the relative intensity of PI fluorescence increases with the dose delivered for three cell models: 2D cell monolayer, 3D spheroids, and 3D GelMA encapsulated cells. Higher PI intensities were observed in MRT valley doses relative to its SBB dose counterparts due to the presence of a high peak dose. Variability of PI intensity was observed in 3D tumor spheroids (27.22% and 33.11% maximum coefficient of variation (CV) for 9L and U87, respectively) which is caused by its senescent or necrotic core as the culture medium nutrients cannot reach the cells at the core. In addition, inconsistency in spheroid shape contributed to the measurement variability. Glioma spheroids have been reported to be difficult to reproduce due to various cell culturing factors^73^. PI intensity variation was also observed in 3D bioprinted cells (48.95% and 46.41% maximum CV for 9L and U87, respectively)) which can be explained by the presence of multiple layers of cells inside the 3D gel structure. The porous structure of GelMA which allows nutrients to diffuse within the encapsulated cells may also influence the fluorescence intensity. Unlike 3D spheroids, 3D GelMA scaffolding limits the shape and environment wherein encapsulated cells can grow that gives bioprinting a higher spatial control and reproducibility. The 3D geometry of encapsulated glioma mimics the tumor tissue geometry and accurately distributes oxygen and nutrition in 3D TME with GelMA hydrogel as one of the popular bioinks^74^.

#### 3D spheroid diameter measurement

**Fig. 10:**
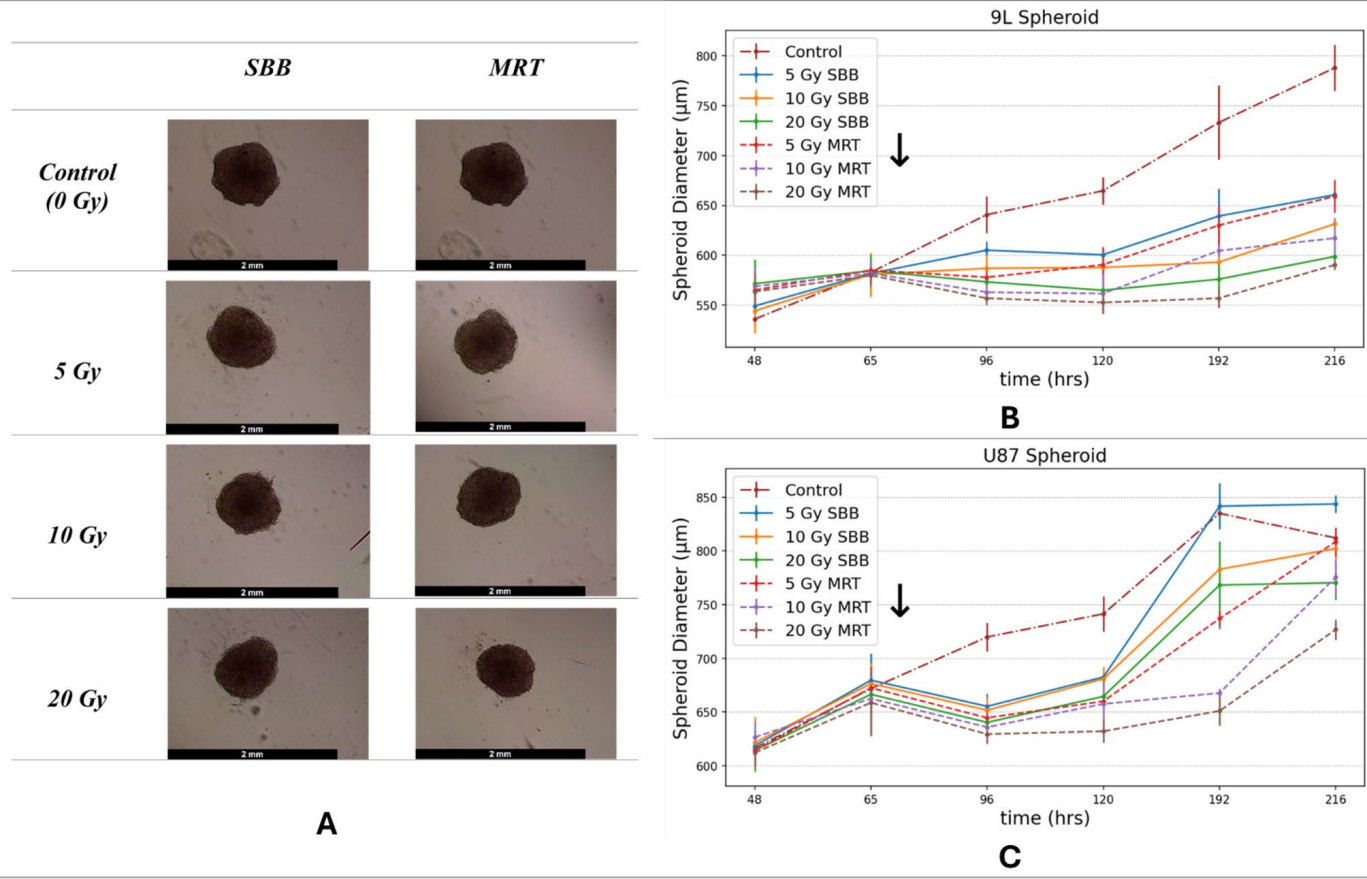
(**A**) Representative brightfield images of spheroids for different doses of synchrotron broad-beam (SBB) and microbeam radiation therapy (MRT) two days after treatment delivery, (**B** and **C**) Line graphs showing the average spheroid diameter ± standard deviation of the mean for different time periods (*n* = 6 for each experiment). Note that the down arrow shown in figures B & C refers to the time of radiation treatment beam delivery.

The induced radiation damage on spheroids was observed after delivering treatment beams by monitoring the decrease in diameter relative to the control samples. Figure 10 shows the decrease in spheroid diameter which started one doubling time after giving treatment. MRT valley doses induced a substantial reduction in diameter relative to its SBB dose counterpart which can be attributed to the high peak dose of MRT beams. These diameter measurements are consistent with the results from the PI intensity measurements which shows MRT produced more cell killing than SBB. A delay in the growth rate of irradiated glioma spheroids relative to the control was also observed. Note that the radiation beams were given once (shown as down arrow in Fig. 10B and **C**, thus the 3D spheroids increased in size after getting smaller due to a single treatment delivery. Multiport MRT beam delivery for all the cancer cell models will be studied in future synchrotron beamtime experiments.

**Fig. 11:**
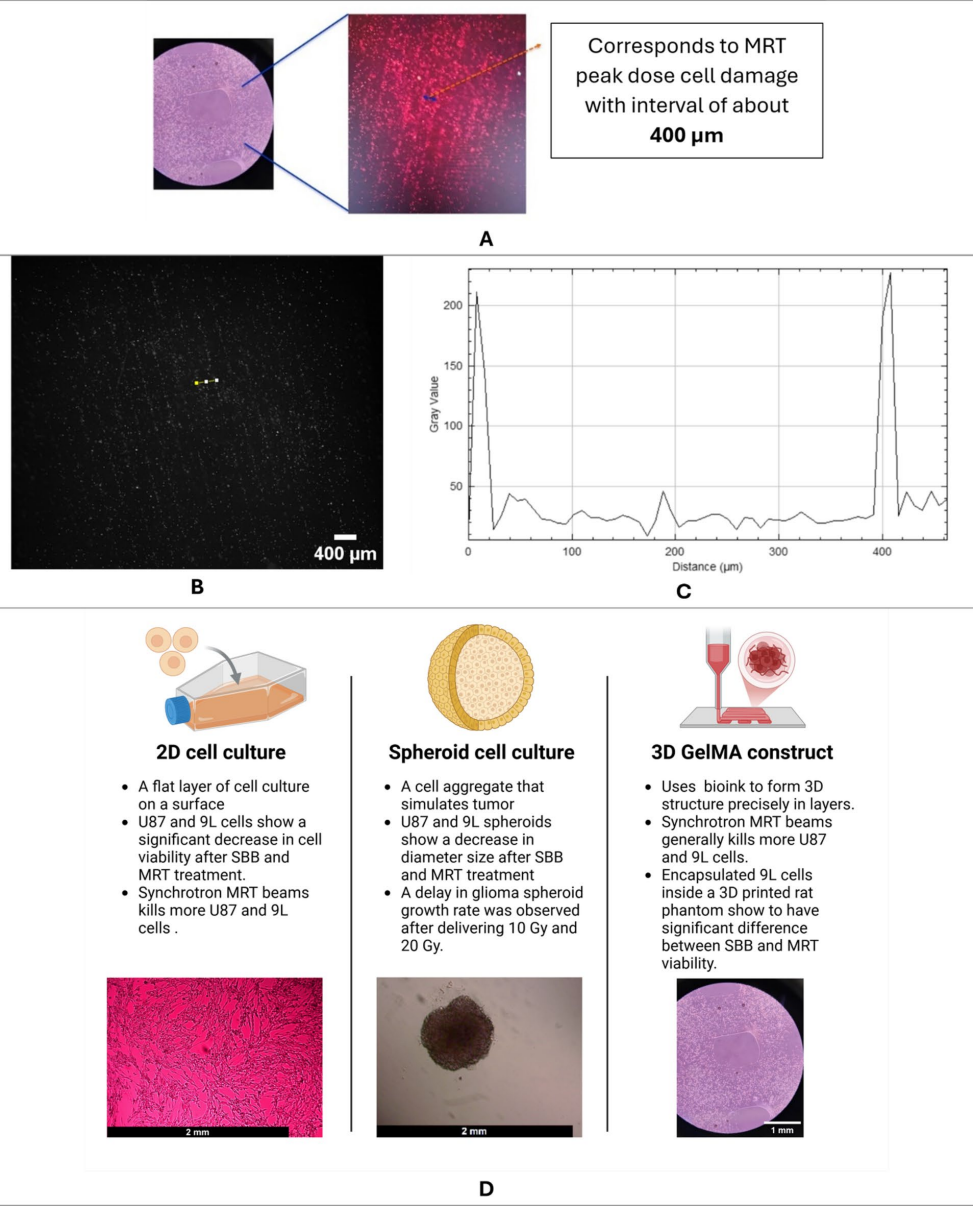
3D bioprinted lattice structure for MRT experiments: (**A**) Brightfield and PI fluorescence imaging of the U87 bioprinted lattice detecting damaged cells due to high peak doses of MRT beam, (**B & C**) Red channel of the PI image and imaging profile showing two gray value peaks due to MRT peak doses, and (**D**) Comparison of three cancer models used in this study. Created in BioRender. Bustillo, J. (2025) https://BioRender.com/l60k787.

### Comparison of cancer models

PI fluorescence imaging shows to be an effective method in evaluating the biological effects of MRT beams. A strong PI signal creating a striated pattern was observed in the 3D bioprinted U87 with 20 Gy valley dose of MRT 120 h after irradiation as shown in Fig. 11. The distance between two strong PI signals (stripe pattern) was measured to be approximately 400 nm which corresponds to the center-to-center distance of MRT peaks. This shows that the spatial distribution of cell damage induced by MRT can be measured using both the 3D bioprinted construct and fluorescence microscopy. The use of sensitive assays such as γH2AX and a confocal microscopy system demonstrated to be able to verify the physical PVDR of MRT can be done in 3D bioprinted constructs as reported in previously published study^75^. This bio-dosimetric method will be done in future studies to analyze the double-strand break in the 3D bioprinted samples after delivering SBB and MRT.

Figure 11.**D** shows a summary of the observed biological responses for all the cancer cell models used in this study. One common observation is that synchrotron MRT mostly kills more glioma cells relative to SBB. An advantage of using 3D bioprinted tumor contructs includes its capability of visualizing MRT damage using fluorescence microscope. In addition, it was observed that 3D bioprinted 9L glioma constucts placed inside a 3D printed rat phantom have significant more cell killing in delivering MRT beams compared to SBB treatment due to high measured PVDR.

## Discussion

In this study, a unique 3D bioprinting protocol using GelMA hydrogel and a compact 3D REDI bioprinter has been characterized in studying the biological effects of synchrotron MRT for glioma cell lines using the instrumentation available at the Australian synchrotron. Gliomas are highly malignant central nervous system tumors and are generally incurable regardless of the current advancements in medicine. Due to its inherent resistance to chemo- and radiation therapy, it demands novel treatment techniques such as delivering spatially fractionated synchrotron MRT beams^10^. Conventional radiotherapy for glioma involves an accumulated dose of 45–60 Gy with 1.8 to 2 Gy per fraction delivered around 5–6 weeks. In addition, in vitro studies for glioma commonly deliver up to 10 Gy^76^. Synchrotron MRT was used in this study as it can deliver higher dose than conventional radiotherapy to the tumor while sparing healthy tissues due to its UHDR and spatial fractionation^14^.

The extrusion bioprinting technique used in this study deposits cell-laden bioink onto a surface using pneumatic or mechanical pressure following the preprogrammed machine instructions to spatially generate the desired structure. Prior to 3D bioprinting of experimental samples, various printing interrelated parameters were optimized such as syringe diameter, bioink viscosity, temperature, and extrusion speed considering the printing resolution and cell viability. These factors contribute to the pressure experienced by cells in the bioink which can negatively affect their cell viability as the shear stress becomes higher^41^. The printability index was used to evaluate if the bioprinted lattice structure is acceptable, and repeatable. In addition, a GelMA concentration of 8% w/v was chosen as it gives a good repeatable lattice structure, high cell viability, and cell spreading as recommended by published studies^44,49,50^. The hydrogel matrix stiffness is another important factor to consider as it influences crosslinking, printability, and cell viability^40^. Furthermore, external factors such as temperature, and humidity during bioprinting inside a biosafety cabinet can influence the outcome of the bioprinted construct. Thus, repeating the bioprinting optimization prior to an experiment is important. Achieving an optimal 3D bioprinted construct can be challenging and various optimization methods have been proposed including the use of machine learning^51^.

MRT glioma survival is shown to be correlated to the SBB conditions using the valley dose as suggested by published studies and was used in this study^10,77^. Both the monolayer and 3D GelMA U87 and 9L cells show to have significant differences in their cell viability after delivering doses of SBB. This difference in radiotherapy biological response is attributed to the differences in cell-cell interactions, and accessibility to nutrients and oxygen between 2D monolayer and 3D GelMA culture. On the other hand, only the 10 Gy MRT for U87 was seen to have a significant difference in the cell viability between the glioma monolayer and 3D GelMA for MRT experiments. Future studies should consider checking the viability after more than 72 h. post-irradiation (long-term survival) to consider the effect of lower valley doses on cell death and bystander effects. Lastly, placing the glioma samples inside a phantom (rat and human head) significantly affects the difference between SBB and MRT doses. Smaller phantoms such as a rat phantom produces high PVDR at the sample location, making MRT more effective in killing glioma cells as shown in Fig. 8.**F**.

PI fluorescence imaging was shown to be useful in evaluating the extent of cell kill after delivering SBB and MRT beams of different doses for monolayer, spheroid, and 3D GelMA. The trend of PI signal is similar with the measured cell viability using WST-1 assay. One advantage of using PI staining imaging is its capability to visualize and to measure the cell damage due to high peak doses. The 3D bioprinted GelMA can resolve the lattice pattern of MRT which is a promising application in correlating 3D cell damage to the spatially fractionated MRT dosimetry. This method of visualizing cell damage can also be studied and used for other SFRT modalities such as GRID, Lattice, and minibeam radiation therapy.

Furthermore, the 3D spheroid diameter was shown to significantly reduce from the control group. Higher radiation doses (10 Gy and 20 Gy) caused a delay in the glioma spheroid growth rate. This can be a reliable method in characterizing spheroids aside from PI imaging.

Overall, 3D bioprinting is a cutting-edge technique that enables the creation of 3D cell models that mimic in-vivo characteristics better given its 3D structure, and scaffolding. The bioink can act as the ECM or TME for a more complex cancer model, and the bioprinter can be used to arrange the cell in a 3D space based on a computer-aided design (CAD) model (such as a lattice). The result of this study shows that 3D GelMA has different radiobiological responses relative to monolayer and spheroid. It was also reported in literature that 3D bioprinted glioma models have different radioresistance characteristics compared to 2D cell model due to the expression of radiation tolerance gene^31,33^. Due to the pores of the GelMA hydrogel and the bioprinted lattice structure, the encapsulated cells can access oxygen, and nutrients through diffusion which is a problem in big spheroids. The flexibility of 3D bioprinting in precisely placing layers of bioinks based on medical images (i.e. CT or MRI) makes it more promising in medical physics in modeling patient specific tumor targets^39^. Future research should investigate the effect on the cell’s DNA double-strand breaks using methods like γH2AX after delivering SBB and MRT beams to the samples. This could also be used to study biological dose response of spatially fractionated beams such as MRT^75^. In addition, the X-ray FLASH effect can be investigated by co-culturing cancer cells with healthy cells following the 3D bioprinting protocol presented in this study. This would be interesting to detect the possible reduction in normal cell damage after delivering UHDR of SBB and MRT.

## Conclusion

This study presented a 3D bioprinting protocol using a compact 3D REDI bioprinter machine, GelMA hydrogel, and glioma cells (U87 and 9L) for experimental microbeam radiation therapy at the Australian synchrotron. The biofabrication of glioma tumor construct simulates some aspects of a real TME which offers a good 3D cancer cell model to study the efficacy of radiation treatment. The radiobiological characterization of 3D bioprinted glioma construct relative to monolayer and spheroid was presented by delivering various doses of treatment beams using SBB and MRT. Synchrotron dosimetry, and Monte Carlo simulation were performed prior to biological experiments. The use of 3D printed rat and human phantoms was shown to be an effective irradiation setup to simulate scattering conditions present in clinical and in vivo radiation treatment delivery important in MRT and other SFRT as it directly affects the PVDR. Cell viability assay, spheroid diameter monitoring, and fluorescence imaging were shown to be effective methods in measuring the effects of radiation dose delivery.

The use of 3D bioprinted GelMA cell construct shows to have some different treatment responses relative to its monolayer and spheroid counterpart. Moreover, the striated pattern of dead cells due to MRT beam delivery can be visualized using 3D bioprinted glioma tumor constructs. This study emphasizes the strategies and advantages in using 3D bioprinting techniques in creating in vitro models that can mimic the conditions of an in vivo counterpart. Furthermore, the study’s results are the initial steps in developing a more complex 3D cell culture model which is composed of healthy cells to consider the normal tissue sparing of MRT while maintaining good tumor control. With the rapid development in bioprinting, the addition of a controllable blood vessel network is another interesting extension of this research that will allow a more realistic in vivo biological response.

## Data Availability

All data are available within this manuscript.
